# Effect of a Secure Messaging System on Workplace Violence at Sinai Chicago

**DOI:** 10.7759/cureus.94055

**Published:** 2025-10-07

**Authors:** Furkhan Ali, PrinceEli H Banner, Mohammed A Yousef, Karen Orenshteyn, Sudeekshya Acharya

**Affiliations:** 1 Internal Medicine, Mount Sinai Hospital, Chicago, USA

**Keywords:** medical workplace violence, psychological impact of workplace violence, secure messaging system, workplace violence, workplace violence prevention

## Abstract

Workplace violence in healthcare settings is a well-recognized hazard that includes verbal abuse, threats, and physical assaults. This affects both employees and the quality of care provided. Exposure to workplace violence can lead to employee stress, burnout, and resignation. Quality of care suffers due to providers avoiding violent patients, increased use of security, increased utilization of restraints, and stress to adjacent patients witnessing violence. Healthcare providers working in emergency departments, critical care units, psychiatric units, and trauma centers are particularly vulnerable to violence. This violence is not only from patients, but also from their families. This study evaluates the effect of implementing the Tiger Connect secure messaging system on workplace violence incidents reported by the security department at the Sinai Chicago health system. Data was collected from the security department at the Sinai Chicago health system between March 1st and October 31st, 2024. A paired t-test showed a statistically significant decrease in workplace violence incidents (p-value = 0.027), with a 42.4% reduction in the number of incidents from 356 to 205 (95% CI: 8.11-67.35). This implies that the chance of this reduction happening due to chance alone is less than 5%. To our knowledge, this is one of the first studies to quantitatively assess the impact of a secure messaging system on workplace violence in healthcare. This article is unique in that it analyzes the quantitative effect of a secure messaging system on the incidence of workplace violence in healthcare. Secure messaging can facilitate rapid and effective communication. This can result in faster response times, improved coordination, and earlier intervention in violent situations. This study highlights the potential of secure messaging systems to promote a safer and more supportive work environment. Further research can delve into qualitative variables in workplace violence incidents, interventions besides a secure messaging system, and the effects of a secure messaging system on other institutions on workplace violence.

## Introduction

Violence against healthcare workers is a global concern that has the potential to threaten the integrity of healthcare delivery [1]. Healthcare workers are increasingly subjected to verbal, psychological, and physical violence. This violence is not only perpetrated by patients, but their families as well. This is particularly true in high-stress environments such as emergency departments, psychiatric units, and intensive care settings. Healthcare workers face verbal abuse, physical assault, and threats. For quite some time, workplace violence has been a recognized problem in the healthcare sector; the COVID-19 pandemic intensified this problem. Frontline workers were perceived as unreliable due to their inability to provide tangible solutions in the face of overwhelming system failures, beyond their grasp, including low staffing ratios, long wait times, lack of hospital beds, inadequate PPE, and high patient mortality rates; this combination created an incubus for public frustration [2-4]. Unfortunately, the true number of healthcare violence incidents may never be truly known given that many staff members do not report incidents due to the belief that nothing will change, fear of retaliation, and inadequate reporting systems [2]. The increased incidents of verbal and physical abuse during the pandemic are seen; this trend has been observed globally, not only in the United States [5-7]. This prevalence of violence prompts the need for interventions to curtail and/or eliminate workplace violence against healthcare workers, including but not limited to security training seminars and the establishment of secure messaging systems across hospital networks. One of these secure messaging systems, Tiger Connect, was probed during this study. Without a secure messaging system, staff at many organizations rely on intercom alerts and the physical presence of security personnel to mitigate violence. Oftentimes, violence has already occurred or continues to be in process by the time security personnel arrive. A secure messaging system, like Tiger Connect, allows security officers to arrive in a more timely fashion and de-escalate or contain violence quickly. Therefore, the use of Tiger Connect was studied in this project. Other attempts to decrease workplace violence at Sinai Chicago were implemented during this investigation; however, only the use of a secure messaging system was analyzed during this study due to its efficacy in improving security response times and value in timely de-escalation. The objective of this study was to evaluate whether the implementation of the Tiger Connect secure messaging system reduced the incidence of workplace violence against staff at Sinai Chicago using a pre-post analysis comparing data four months before and four months following July 1, 2024.

## Materials and methods

Study design

This was a retrospective post-intervention study that compared the incidence of workplace violence at Sinai Chicago before and after implementation of a secure messaging system. Sinai Chicago consists of two main hospital sites: Mt Sinai Hospital and Holy Cross Hospital. Mt Sinai Hospital is a safety net hospital with 319 beds [8]. In addition, it serves as a level one trauma center [8]. It provides several healthcare services through different departments. These departments include, but are not limited to, Oncology, Cardiology, Endocrinology, Otolaryngology, Hand Surgery, Ophthalmology, Orthopedics, Rheumatology, and Wound Care [8]. The other hospital site of Sinai Chicago, Holy Cross Hospital, is a community hospital with 264 beds [9]. This hospital also provides many different services through its diverse departments. These departments include, but are not limited to, Psychiatry, Oncology, Cardiology, Endocrinology, Gastroenterology, Surgery, Infectious Disease, Podiatry, Rheumatology, Sleep Medicine, Substance Abuse, Urology, and Wound Care [9]. Both of these institutions work to serve thousands of patients each year in some of the most underserved areas in Chicago, Illinois [8,9].

**Figure 1:**
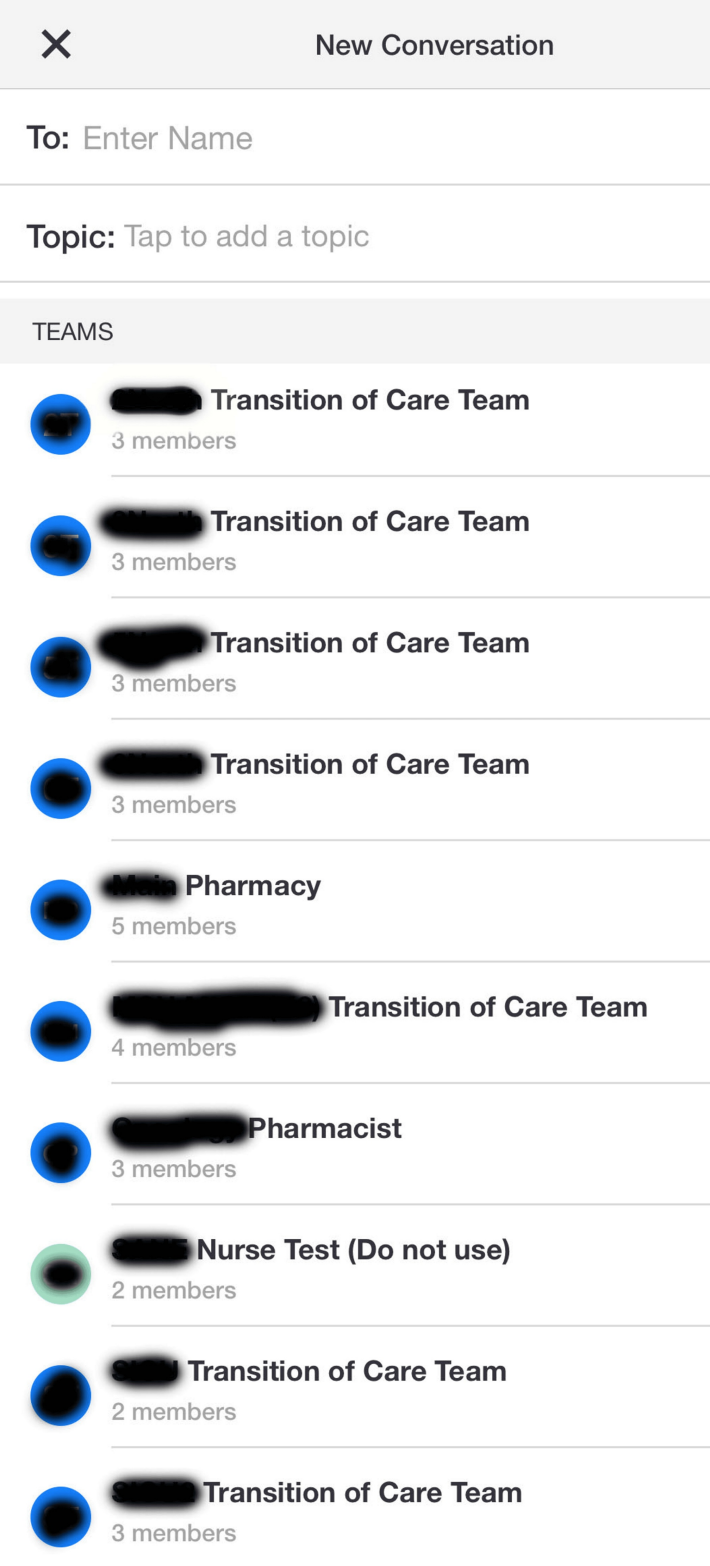
Tiger Connect Search Feature

**Figure 2:**
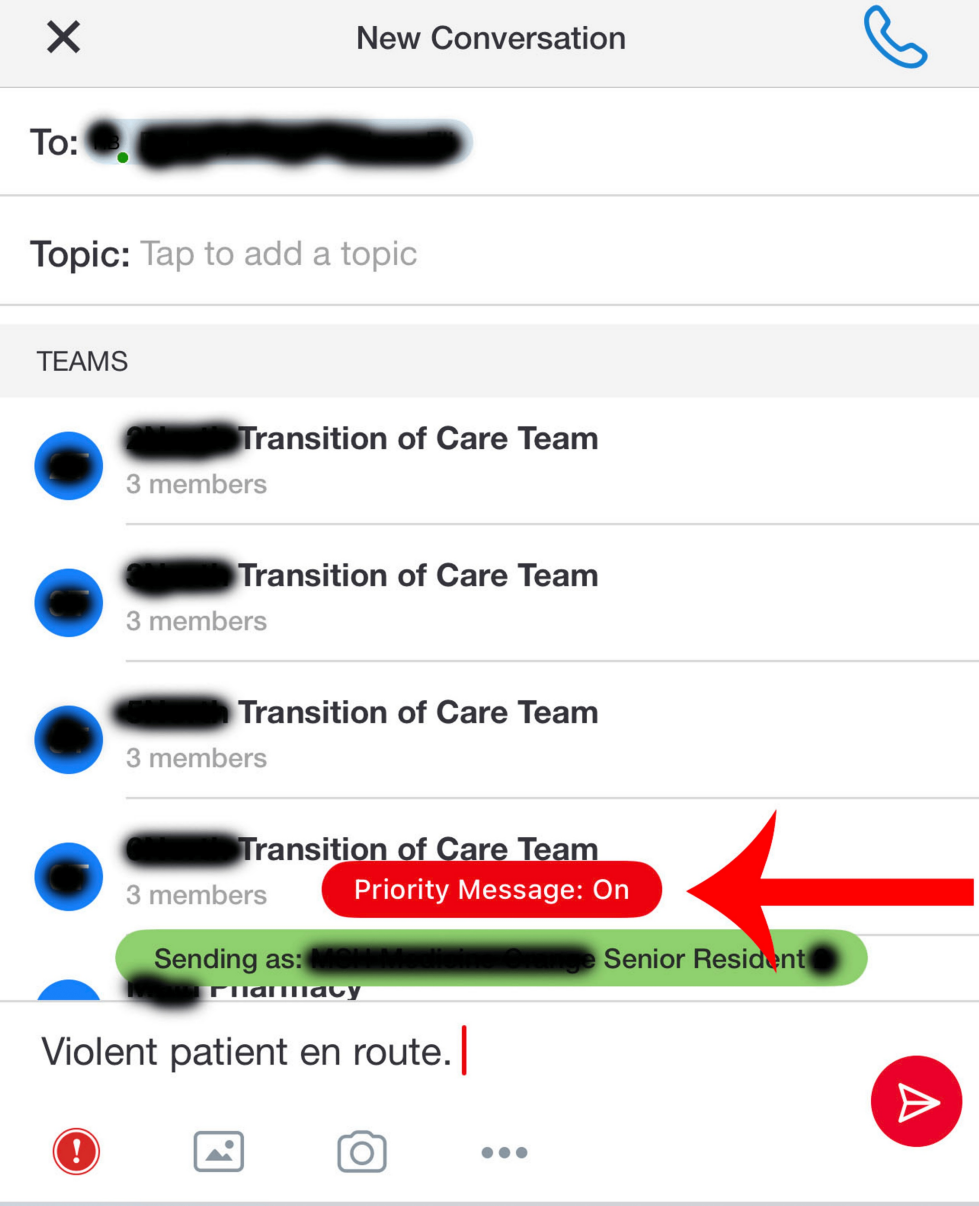
Tiger Connect Priority Message Feature

Tiger Connect is a secure messaging platform that facilitates pre-arrival information sharing, staff collaboration, and timely event notifications [10]. It serves as a messaging platform between healthcare facility employees through the Tiger Connect app [10]. Staff members need to log into the app on their phone or mobile device. Then they can search for other users and message one or more of them directly (Figure 1). Moreover, the messaging system has a priority feature to send urgent messages which bypasses silent and do not disturb modes on mobile devices (Figure 2). This messaging system was adopted to help improve communication and response times. Anecdotal concerns voiced by staff included poor response times to violent situations despite several warning signs and poor communication between healthcare staff. This may have been a factor in the adoption of Tiger Connect by the security department. This study chose to compare the incidence before and after adoption of this messaging system using a paired t-test. A paired t-test was chosen due to the data groups being from the same organization before and after the intervention.

Study population and sample size

The population examined in this study was the staff at Sinai Health System in Chicago. Sinai Chicago employs over 3500 individuals [11]. Staff perform various roles and have different interactions with patients and family members. Staff in all departments with access to the Midas system were included. Staff excluded from the study include those without computer access, those with computer access who cannot fill out Midas reports, and those who were unable to fill out Midas reports documenting violent situations they experienced or observed. This affects the generalizability of the findings in this study because there could be many more incidents of workplace violence involving staff excluded from this study, which could not be accounted for because they were not reported in the Midas system. 

Study measures

**Figure 3:**
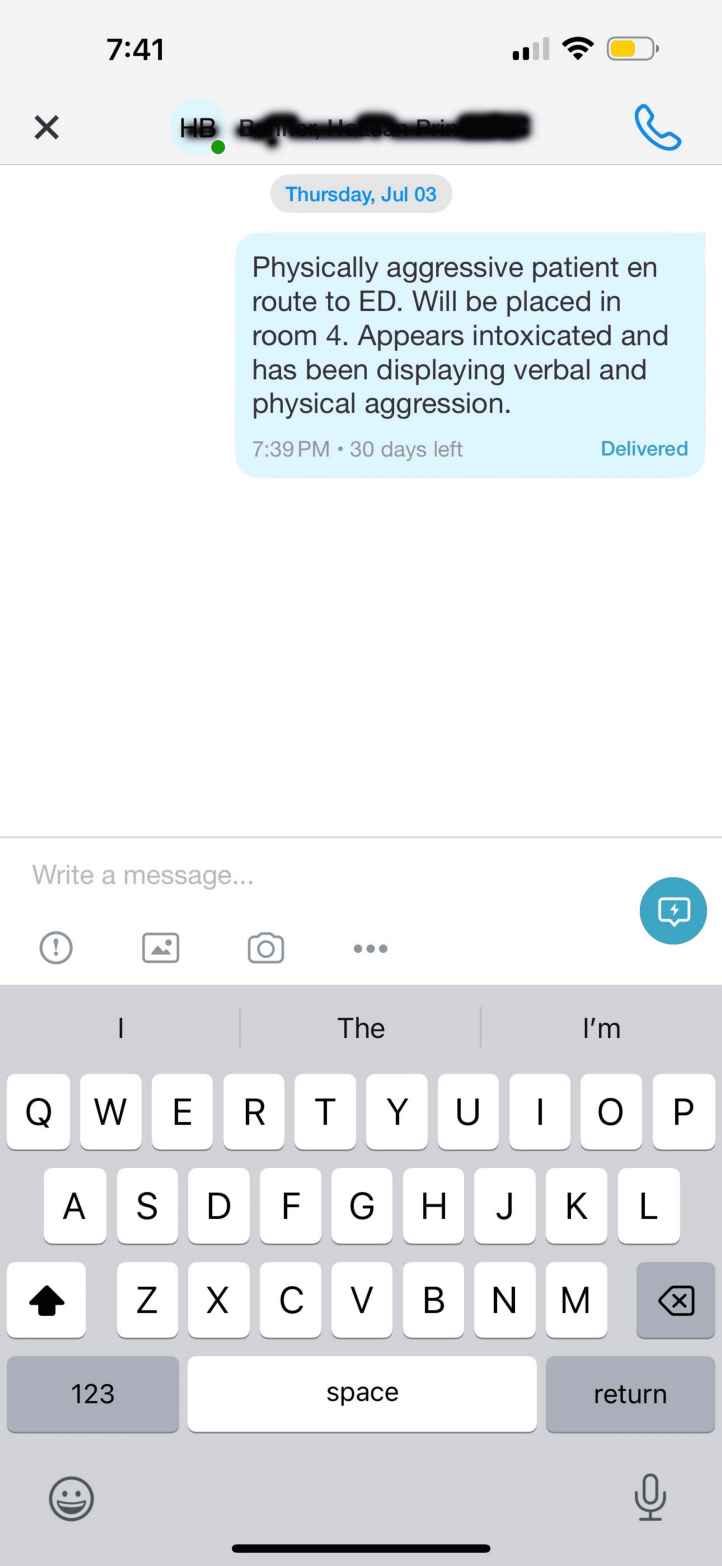
Tiger Connect Message Example

Researchers for this study performed a literature review of articles regarding workplace violence. Standardized criteria for designating an incident as workplace violence could not be found. Nevertheless, the Occupational Safety and Health Administration (OSHA) defines workplace violence as "any act or threat of physical violence, harassment, intimidation, or other threatening behavior that occurs at the work site" [12]. Data regarding incidents for this study was obtained from the Mt Sinai Hospital Chicago Security Department. Only Midas reports tagged as workplace violence were included. Data extraction was automated. No filters or keywords were provided or included in the data set. Interventions to decrease workplace violence implemented at Sinai Chicago were provided as well. In addition to the systemwide adoption of Tiger Connect by the Mt Sinai Hospital Chicago security department, other interventions included increased security patrols in the behavioral health and emergency department units along with safety presentations at new hire orientations. These confounding variables were not accounted for in the analysis. The intervention of adopting the Tiger Connect messaging system across the security department was finalized on July 1st, 2024. Tiger Connect provides a channel for employees in healthcare settings to rapidly disseminate information. In addition, the platform supports improved efficiency, clinical collaboration, and responses to alerts and alarms [10]. This allows security personnel to communicate with clinical providers and each other to de-escalate and contain violent situations. Moreover, the platform supports EMS providing information to healthcare facilities prior to patients even arriving at a healthcare site [10]. This allows security personnel to be notified of potentially violent individuals before they even arrive. All in all, Tiger Connect provides a convenient and efficient way to send information to other healthcare staff (Figure 3).

Tiger Connect has been present at Mt Sinai Hospital Chicago since January 2023. However, July 1st, 2024 saw the complete adoption of this messaging system by the security department. The number of incidents from March 2024 to October 2024 was obtained. This time frame was chosen since data for an equal number of months before and after the implementation of Tiger Connect across the security department at Mt Sinai Hospital Chicago was available using this time frame. Data stratifying some months by different Sinai Chicago locations was provided as well. This stratification was not used in the final analysis and was only for descriptive purposes. The data was then organized into a table. Afterwards, the number of workplace violence events four months prior to July 1st, 2024 and four months after was compared. The intervention date of July 1st, 2024 was chosen due to the complete adoption of Tiger Connect by all officers in the security department. 

Ethics statement

IRB approval was not required for this study due to no identifying information being obtained. Initially, an application for IRB approval was started. During this process, the project was discussed with the Health Services Research Director. Initial brainstorming of the project yielded plans to obtain specific identifying details regarding workplace violence incidents. As the protocol was finalized, these plans were abandoned. Therefore, the Health Services Research Director at Mt Sinai Hospital Chicago determined that IRB approval was not necessary for this study.

Statistical analysis

An alpha value of 0.05 was set before statistical analysis. Normality and equal variance were not checked. This data was then analyzed by a paired t-test using Jamovi software version 2.6.26 (Jamovi, Sydney, Australia). A paired t-test was used because data was collected for the same health system before and after an intervention. Assumptions like normality and equal variances were not tested. Jamovi software was chosen because it was the first free software application for statistical analysis recommended to the research team. Jamovi is a statistical software with several capabilities. It can run multiple tests including t-tests, ANOVAs, correlation tests, nonparametric tests, contingency tables, regression tests, reliability analyses, and factor analyses [13]. 

## Results

A sample size of 561 workplace violence incidents between March and October 2024 was used. No assumptions, such as normality or equal variance, were tested prior to analysis. A paired t-test comparing the number of workplace violence incidents at Sinai Chicago before and after the adoption of the Tiger Connect secure messaging system yielded a t statistic of 4.054 and a p-value of 0.027 (Table 1). Therefore, the analysis yielded a statistically significant decrease in workplace violence at Sinai Chicago following the implementation of the Tiger Connect secure messaging system by security personnel. In addition, the analysis resulted in a confidence interval (CI) of 8.11-67.35 (Table 1). Although the CI (8.11-67.35) indicates variability in the effect size, the result remains statistically significant.

**Table 1:** Paired T-test Analysis Comparing Incidence of Workplace Violence at Sinai Chicago in March-June versus July-October

Parameter	Value
T statistic	4.054
p-value	0.027
CI	8.11-67.35

**Figure 4:**
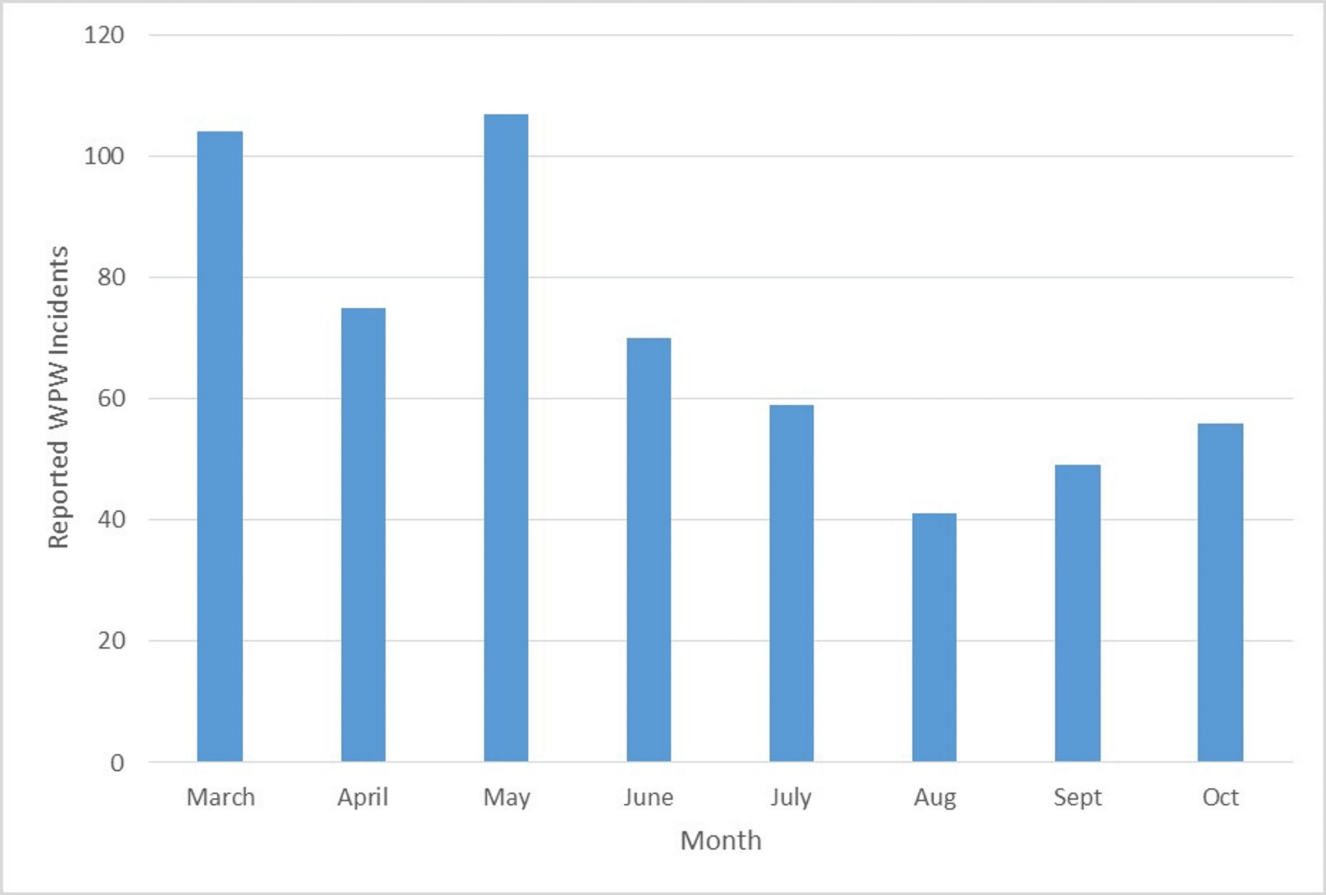
Midas Reported Workplace Violence at Sinai Chicago in 2024 WPW: Workplace violence

In the four months prior to July 1st, 2024, 356 workplace violence incidents were reported across Sinai Health System. This number decreased to 205 during the four months after July 1st, 2024. This represented a 42.4% decrease in workplace violence incidents reported. These changes are illustrated below (Figure 4, Table 2). This is clinically significant because the number of violent incidents decreased by over 150 in this four-month period after implementing Tiger Connect. This fostered a safer work environment and allowed for more effective patient care. 

**Table 2:** Workplace Violence Events at Sinai Chicago in 2024 WPW: Workplace violence

WPW Events 2024	
Month	#Events
March	104
April	75
May	107
June	70
Total Jan-June	356
July	59
Aug	41
Sept	49
Oct	56
Total July-Oct	205

## Discussion

The results of our study suggest that implementation of a secure messaging system for security personnel may be associated with a decrease in workplace violence reported across Sinai Health System. According to our data, there was a 42.4% decrease in workplace violence incidents reported across Sinai Health System after adopting Tiger Connect. Usage of this secure messaging system allowed for quick and efficient communication between the security personnel and prompt action in resolving incidents. Specific qualitative examples/feedback could not be obtained from security personnel due to confidentiality. Moreover, use of the secure messaging system allowed security to be notified of potentially violent incidents. This allowed them to arrive and diffuse situations prior to them escalating. Additionally, it was anecdotally observed that WPV due to patients/clients/visitors saw the largest drop in incidents after adoption of the messaging system. 

A review of prior research regarding workplace violence in healthcare yielded some themes. Review of documented literature illustrates workplace violence happens on a regular basis at healthcare institutions worldwide [1-7,14-21]. In addition, workplace violence has been going on for years. Despite the evidence of multiple violent incidents afflicting healthcare workers, few studies have monitored the effects of interventions on the incidence of workplace violence in healthcare settings. Moreover, most studies in the literature are qualitative in nature [2-6,14-16]. Most studies analyzed factors involved in violent incidents. Some qualitative studies provide suggestions to potentially reduce workplace violence [6,14,15]. Very few studies are quantitative in nature [15-19]. Even fewer of these studies analyzed the incidence of workplace violence [15,17]. Cai et al. conducted an analysis of workplace violence before and after educating nurses about violent situation prevention [17]. Researchers found a 17.7% decrease in the incidence of workplace violence against nurses at a hospital in Suzhou, China [17]. A study by Somani et al. did a systematic review of interventions to decrease workplace violence against nurses [15]. However, both of these studies only analyzed workplace violence against nurses. Of these studies, none analyzed the implementation of a secure messaging system by security personnel. Therefore, this study stands out as the only quantitative study to analyze the effects of secure messaging system adoption by security on the incidence of workplace violence in a healthcare setting. Moreover, this study is unique in that it analyzes the incidence of workplace violence against all hospital system workers, including, but not limited to, nurses, doctors, technicians, transporters, and custodians.

We utilized a paired t-test for statistical analysis and found statistical significance with a p-value of 0.027. The CI was determined as 8.11-67.35. The prevalence of workplace violence incidents still remains high. The International Labor Organization (ILO) did a survey in 2022 and found that almost 23 percent of employees have experienced workplace violence at some point [22]. In 2019, the ILO developed the Convention 190 (C190): a document affirming that workplaces should be completely free from violence [23]. While technological resources appear to be impactful in countering violent incidents, more methods need to be explored and adopted to further decrease the incidence of violence in healthcare settings [2,6,19]. Reinforcing awareness with resources such as notes, posters, and pamphlets throughout the hospital may serve as a deterrent against the occurrence of workplace violence incidents. Moreover, regular conferences for healthcare workers on workplace violence education can help workers deal with incidents as well as reiterate professional conduct in managing such situations. 

Nevertheless, caution is warranted before assuming causality with these findings. The study is limited by sample size as well as some confounding variables. The study took into account only those incidents that were reported with the MIDAS reporting system. Workplace violence incidents that were not submitted to MIDAS were not recorded and therefore not accounted for. In addition, these incidents were self reported by personnel. Moreover, the study as a whole was retrospective in nature. The current incidence of workplace violence may have changed since the timeframe of the collected data. Additionally, other measures were adopted by the security department from July 1st, 2024; these may have had a confounding effect on the data. These included increased patrolling in the ED and behavioral health units (Table 3). The security department also started holding a brief presentation at all new hire orientation programs (Table 3). This enabled healthcare workers to be better equipped at handling workplace violence incidents (Table 3).

**Table 3:** Concurrent Interventions to Decrease Workplace Violence at Sinai Chicago

Intervention	Location	Adoption Date	Possible Impact
Increased security patrols	Emergency and Behavioral Health Units	7/1/2024	Improved response times to violent incidents.
Workplace Violence Education during Orientation	Auditorium/ Conference Rooms	7/1/2025	Improved awareness/preparedness to hand violence.

In addition, another limitation of this study is its highly basic design. This study relied on a simple paired t-test to analyze the data collected. Moreover, confounders and qualitative factors within violent incidents were not analyzed. Additional perspectives in relation to violent incidents from victims, security personnel, bystanders, and perpetrators were not taken into account. Moreover, other variables, such as location, timing, and severity of violent incidents, were not studied. Moreover, other methods to obtain more thorough data or analyze the intervention along with other interventions were not utilized. In particular, methodological restraints rely on a simple statistical test analyzing data before and after one intervention for this study. Additionally, data was only obtained from an online reporting system report, which may or may not represent the true number of workplace violence incidents at the organization. Moreover, a lack of a control group after the adoption of the secure messaging system decreases the power of the study. All these limitations and confounding variables limit the generalizability of findings in this study.

Despite these limitations, the findings suggest a significant association between secure messaging implementation and reduced workplace violence. This study encourages the use of a messaging system in order to enhance security efficiency and prevent workplace violence incidents. 

## Conclusions

Despite the prevalence of workplace violence in healthcare, there are efforts being made to eliminate it. Multiple organizations are working on mitigating workplace violence. This study evaluated the impact of implementing a secure messaging system designed to improve communication among security personnel. This study found a 42.4% decrease in workplace violence after adoption of this secure messaging system, which was also statistically significant. To our knowledge, this is among the few studies that provide quantitative evidence on the impact of an intervention aimed at reducing workplace violence in healthcare. Nevertheless, confounding variables like increased patrols and staff presentations during orientation were present. This may also have had an effect on the incidence of workplace violence at Sinai Chicago. Consequently, data from this study provides valuable preliminary evidence that secure messaging systems may contribute to improved workplace safety, but further research is needed to confirm causality and generalizability. Moreover, this study opens the door for further areas of inquiry. These areas include qualitative variables in violent situations, other interventions to prevent violent incidents, and appropriate ways to de-escalate and/or resolve violent situations. Further research can delve into qualitative variables in violent situations through interviews with security personnel. Moreover, interviews with caregivers can be done to obtain staff perceptions on workplace violence. Additionally, interviews with patients who have been involved in workplace violence can be facilitated in an attempt to ascertain their outlook regarding workplace violence incidents. Future studies could replicate this intervention of adopting a secure messaging system in the security department in other healthcare settings to assess its broader applicability and impact. Additionally, future studies can utilize more thorough statistical analyses that account for confounding variables. Unfortunately, workplace violence is a persistent issue plaguing healthcare institutions worldwide. However, targeted interventions, such as secure messaging systems, may contribute to safer work environments, enabling healthcare providers to continue delivering care effectively.

## References

[1] (2025). Preventing violence against health workers. https://www.who.int/activities/preventing-violence-against-health-workers.

[2] Phillips JP (2016). Workplace violence against health care workers in the United States. N Engl J Med.

[3] Yan S, Feng J, Gan Y (2023). Prevalence and predictors of workplace violence against emergency physicians in China: a cross-sectional study. Hum Resour Health.

[4] Lanctôt N, Guay S (2014). The aftermath of workplace violence among healthcare workers: a systematic literature review of the consequences. Aggress Violent Behav.

[5] Devi S (2020). COVID-19 exacerbates violence against health workers. Lancet.

[6] Jeleff M, Traugott M, Jirovsky-Platter E, Jordakieva G, Kutalek R (2022). Occupational challenges of healthcare workers during the COVID-19 pandemic: a qualitative study. BMJ Open.

[7] McKay D, Heisler M, Mishori R, Catton H, Kloiber O (2020). Attacks against health-care personnel must stop, especially as the world fights COVID-19. Lancet.

[8] (2025). Mount Sinai Hospital. https://www.sinaichicago.org/en/find-a-location/results/mount-sinai-hospital/.

[9] (2025). Welcome to Holy Cross Hospital. https://www.sinaichicago.org/en/find-a-location/results/holy-cross-hospital/.

[10] (2025). tigerconnect. https://tigerconnect.com/.

[11] (2025). About us - Sinai Health System. https://www.sinaichicago.org/en/about-us/#:~:text=Our%20clinical%20and%20administrative%20leadership,system%20with%20over%203%2C500%20employees..

[12] (2025). Workplace violence. https://www.osha.gov/workplace-violence.

[13] (2025). Features - Jamovi. https://www.jamovi.org/features.html.

[14] Gillespie GL, Gates DM, Miller M, Howard PK (2010). Workplace violence in healthcare settings: risk factors and protective strategies. Rehabil Nurs.

[15] Somani R, Muntaner C, Hillan E, Velonis AJ, Smith P (2021). A systematic review: effectiveness of interventions to de-escalate workplace violence against nurses in healthcare settings. Saf Health Work.

[16] Sari H, Yildiz İ, Çağla Baloğlu S, Özel M, Tekalp R (2023). The frequency of workplace violence against healthcare workers and affecting factors. PLoS One.

[17] Cai J, Wu S, Wang H, Zhao X, Ying Y, Zhang Y, Tang Z (2023). The effectiveness of a workplace violence prevention strategy based on situational prevention theory for nurses in managing violent situations: a quasi-experimental study. BMC Health Serv Res.

[18] Antão HS, Sacadura-Leite E, Manzano MJ, Pinote S, Relvas R, Serranheira F, Sousa-Uva A (2020). Workplace violence in healthcare: a single-center study on causes, consequences and prevention strategies. Acta Med Port.

[19] (2025). Management of aggressive patient situations. https://www.myamericannurse.com/management-of-aggressive-patient-situations/.

[20] (2025). The Illinois Healthcare Workplace Violence Protection Act: what hospitals need to know. https://www.mwe.com/insights/il-health-care-violence-prevention-act/.

[21] Kumari A, Singh A, Ranjan P (2021). Development and validation of a questionnaire to evaluate workplace violence in healthcare settings. Cureus.

[22] (2025). Violence and harassment at work has affected more than one in five people. https://www.ilo.org/resource/news/violence-and-harassment-work-has-affected-more-one-five-people#:~:text=The%20ILO%20(International%20Labour%20Organization)%20has%20a,or%20older%20in%20121%20countries%20and%20territories..

[23] (2025). C190 - Violence and Harassment Convention, 2019 (No. 190). https://normlex.ilo.org/dyn/nrmlx_en/f?p=NORMLEXPUB:12100:0::NO::P12100_ILO_CODE:C190.

